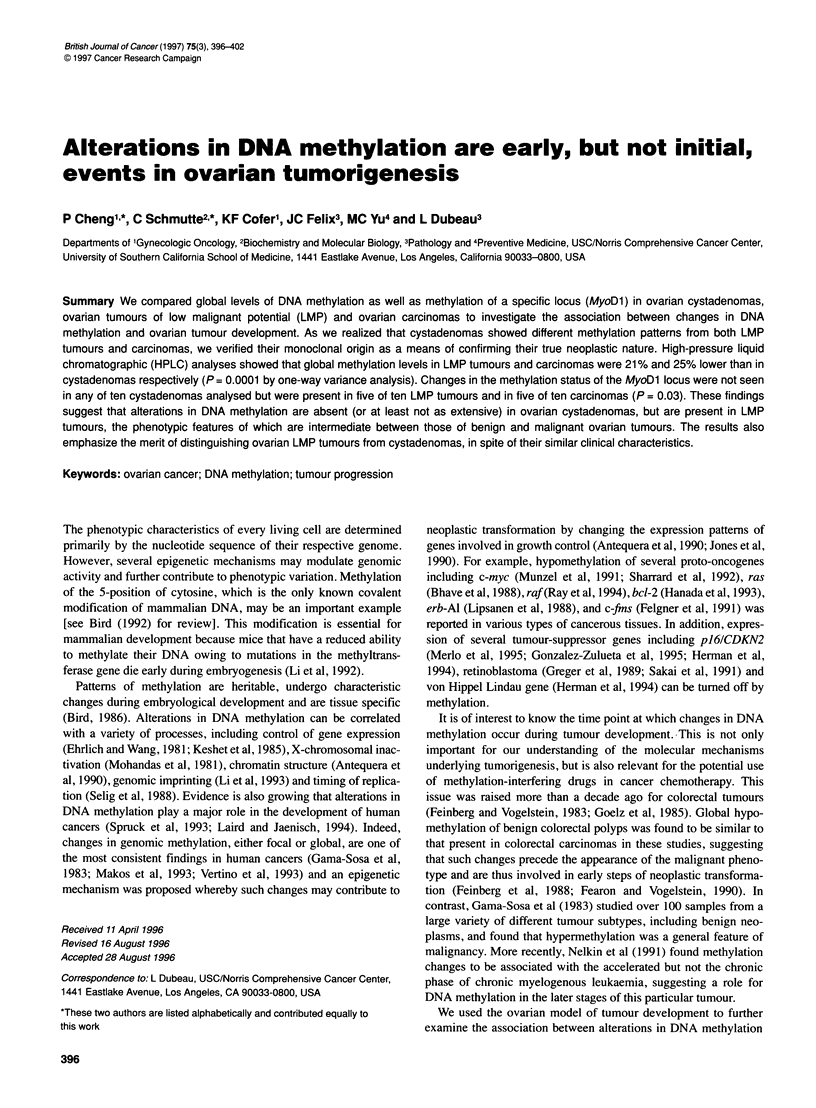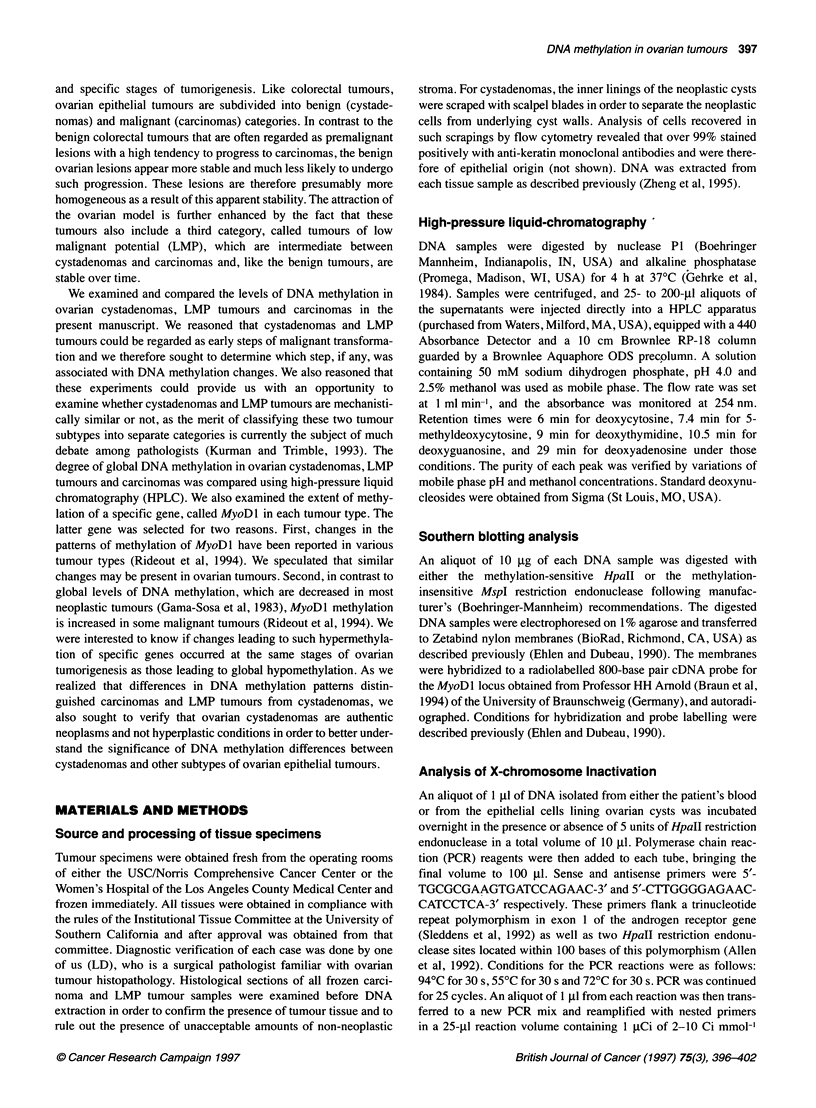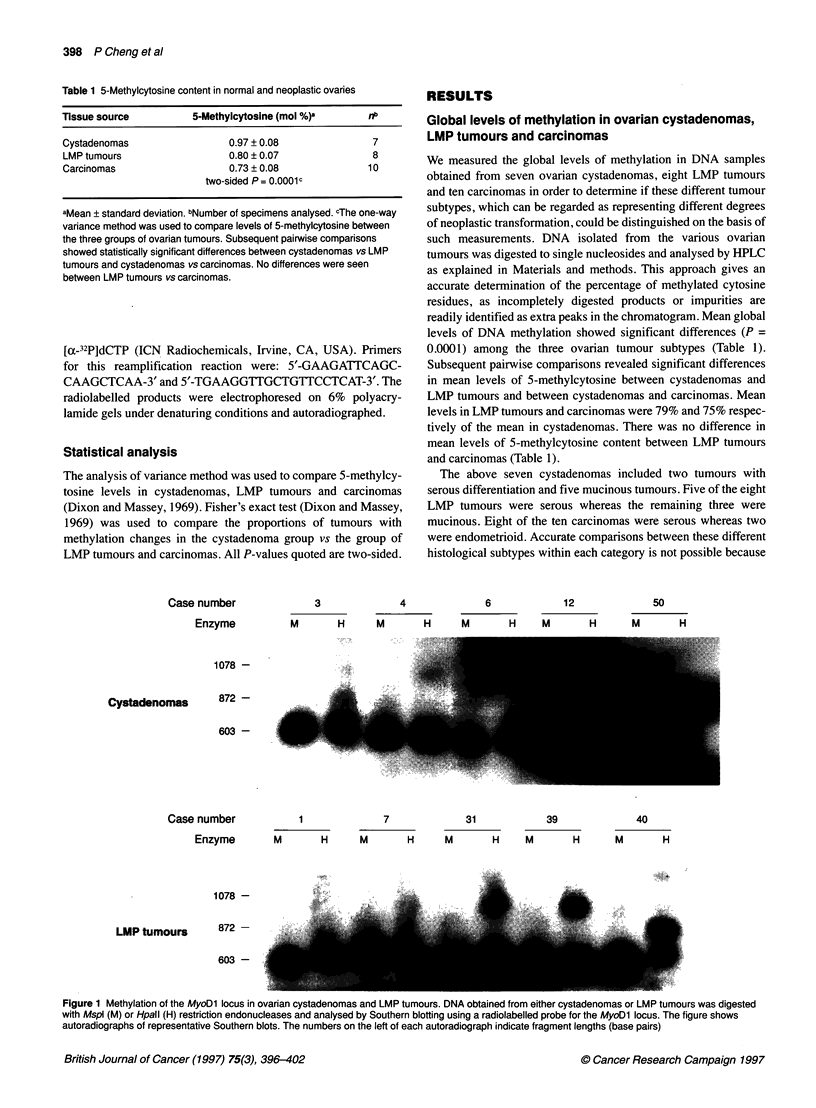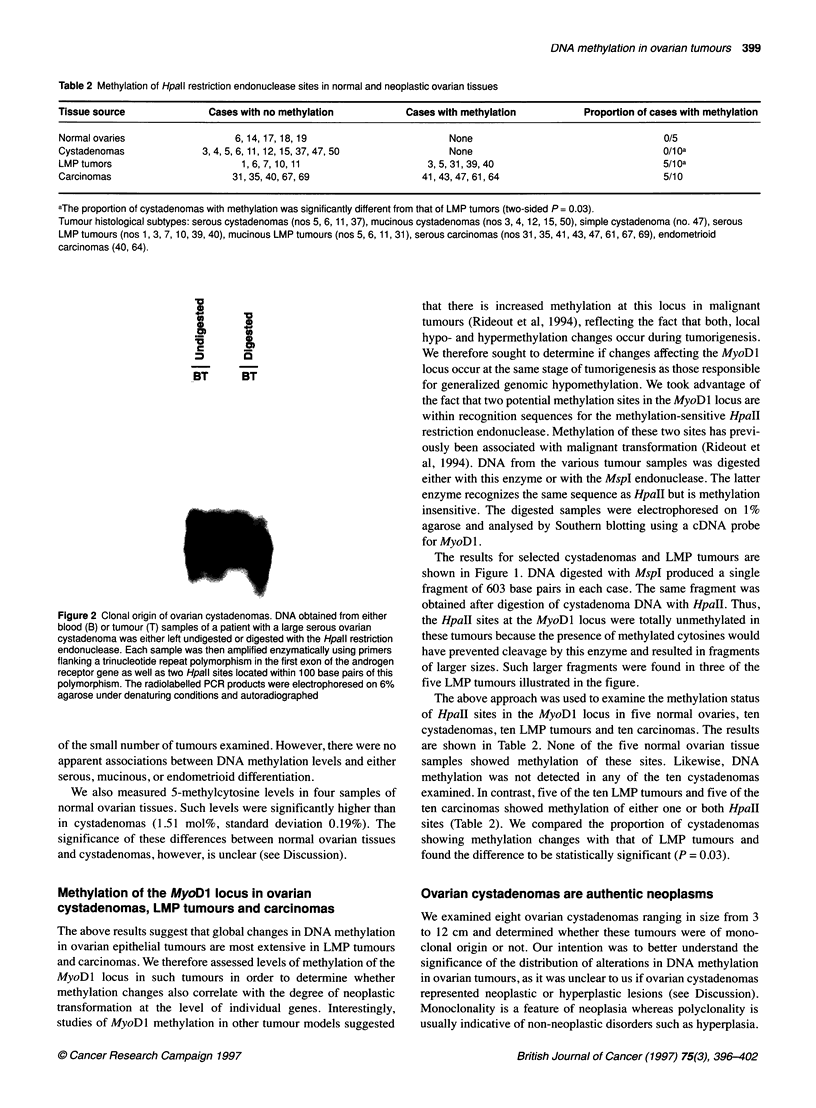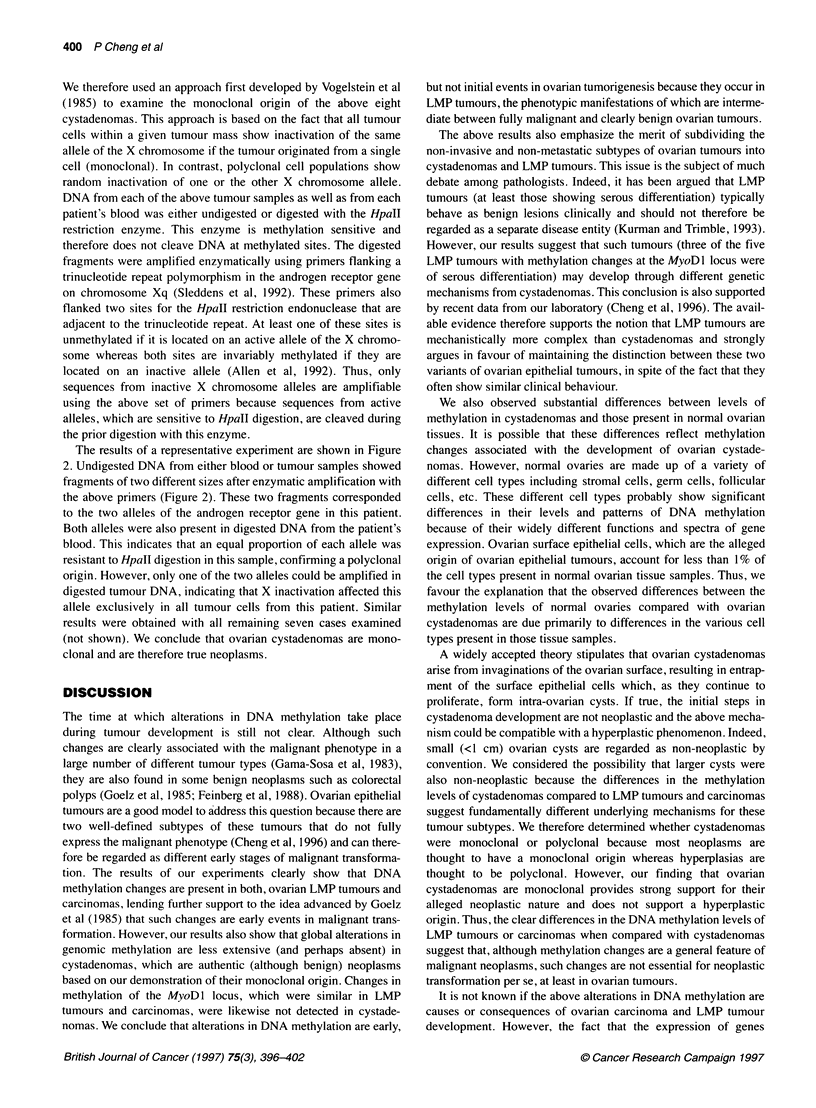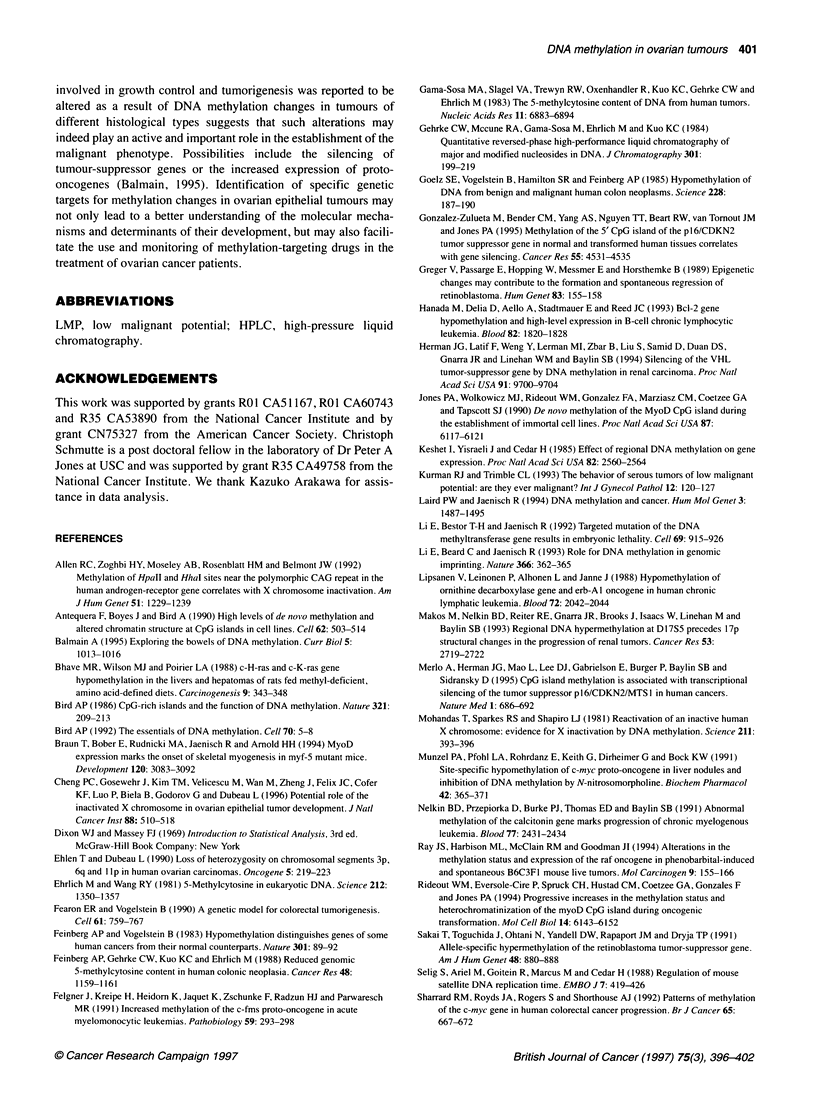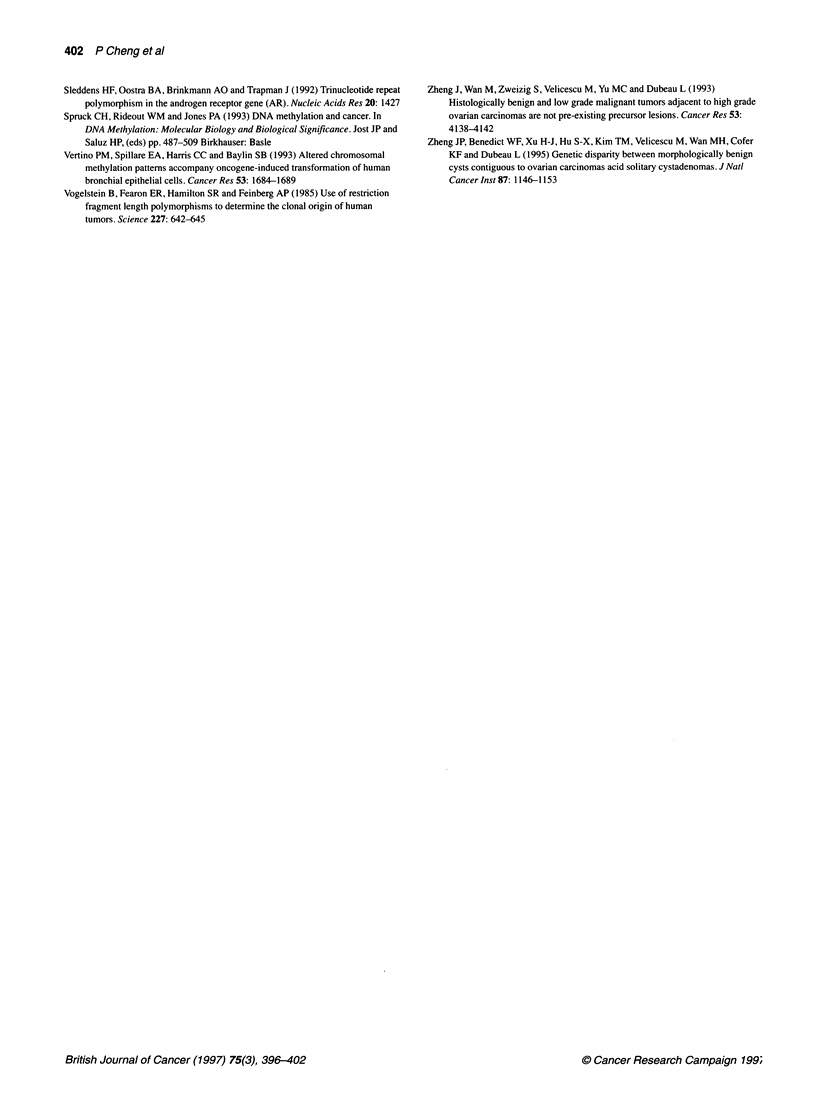# Alterations in DNA methylation are early, but not initial, events in ovarian tumorigenesis.

**DOI:** 10.1038/bjc.1997.64

**Published:** 1997

**Authors:** P. Cheng, C. Schmutte, K. F. Cofer, J. C. Felix, M. C. Yu, L. Dubeau

**Affiliations:** Department of Gynecologic Oncology, USC/Norris Comprehensive Cancer Center, University of Southern California School of Medicine, Los Angeles 90033-0800, USA.

## Abstract

**Images:**


British Journal of Cancer (1997) 75(3), 396–402
© 1997 Cancer Research Campaign

### Alterations in DNA methylation are early, but not initial, events in ovarian tumorigenesis

**P Cheng[1,*], C Schmutte[2,*], KF Cofer[1], JC Felix[3], MC Yu[4] and L Dubeau[3]**

Departments of [1]Gynecologic Oncology, [2]Biochemistry and Molecular Biology, [3]Pathology and [4]Preventive Medicine, USC/Norris Comprehensive Cancer Center, University of Southern California School of Medicine, 1441 Eastlake Avenue, Los Angeles, California 90033–0800, USA

**Summary** We compared global levels of DNA methylation as well as methylation of a specific locus (*Myo*D1) in ovarian cystadenomas, ovarian tumours of low malignant potential (LMP) and ovarian carcinomas to investigate the association between changes in DNA methylation and ovarian tumour development. As we realized that cystadenomas showed different methylation patterns from both LMP tumours and carcinomas, we verified their monoclonal origin as a means of confirming their true neoplastic nature. High-pressure liquid chromatographic (HPLC) analyses showed that global methylation levels in LMP tumours and carcinomas were 21% and 25% lower than in cystadenomas respectively ($P = 0.0001$ by one-way variance analysis). Changes in the methylation status of the *Myo*D1 locus were not seen in any of ten cystadenomas analysed but were present in five of ten LMP tumours and in five of ten carcinomas ($P = 0.03$). These findings suggest that alterations in DNA methylation are absent (or at least not as extensive) in ovarian cystadenomas, but are present in LMP tumours, the phenotypic features of which are intermediate between those of benign and malignant ovarian tumours. The results also emphasize the merit of distinguishing ovarian LMP tumours from cystadenomas, in spite of their similar clinical characteristics.

**Keywords:** ovarian cancer; DNA methylation; tumour progression

The phenotypic characteristics of every living cell are determined primarily by the nucleotide sequence of their respective genome. However, several epigenetic mechanisms may modulate genomic activity and further contribute to phenotypic variation. Methylation of the 5-position of cytosine, which is the only known covalent modification of mammalian DNA, may be an important example [see Bird (1992) for review]. This modification is essential for mammalian development because mice that have a reduced ability to methylate their DNA owing to mutations in the methyltransferase gene die early during embryogenesis (Li et al, 1992).

Patterns of methylation are heritable, undergo characteristic changes during embryological development and are tissue specific (Bird, 1986). Alterations in DNA methylation can be correlated with a variety of processes, including control of gene expression (Ehrlich and Wang, 1981; Keshet et al, 1985), X-chromosomal inactivation (Mohandas et al, 1981), chromatin structure (Antequera et al, 1990), genomic imprinting (Li et al, 1993) and timing of replication (Selig et al, 1988). Evidence is also growing that alterations in DNA methylation play a major role in the development of human cancers (Spruck et al, 1993; Laird and Jaenisch, 1994). Indeed, changes in genomic methylation, either focal or global, are one of the most consistent findings in human cancers (Gama-Sosa et al, 1983; Makos et al, 1993; Vertino et al, 1993) and an epigenetic mechanism was proposed whereby such changes may contribute to neoplastic transformation by changing the expression patterns of genes involved in growth control (Antequera et al, 1990; Jones et al, 1990). For example, hypomethylation of several proto-oncogenes including c-*myc* (Munzel et al, 1991; Sharrard et al, 1992), *ras* (Bhave et al, 1988), *raf* (Ray et al, 1994), *bcl*-2 (Hanada et al, 1993), *erb*-Al (Lipsanen et al, 1988), and c-*fms* (Felgner et al, 1991) was reported in various types of cancerous tissues. In addition, expression of several tumour-suppressor genes including *p16/CDKN2* (Merlo et al, 1995; Gonzalez-Zulueta et al, 1995; Herman et al, 1994), retinoblastoma (Greger et al, 1989; Sakai et al, 1991) and von Hippel Lindau gene (Herman et al, 1994) can be turned off by methylation.

It is of interest to know the time point at which changes in DNA methylation occur during tumour development. This is not only important for our understanding of the molecular mechanisms underlying tumorigenesis, but is also relevant for the potential use of methylation-interfering drugs in cancer chemotherapy. This issue was raised more than a decade ago for colorectal tumours (Feinberg and Vogelstein, 1983; Goelz et al, 1985). Global hypomethylation of benign colorectal polyps was found to be similar to that present in colorectal carcinomas in these studies, suggesting that such changes precede the appearance of the malignant phenotype and are thus involved in early steps of neoplastic transformation (Feinberg et al, 1988; Fearon and Vogelstein, 1990). In contrast, Gama-Sosa et al (1983) studied over 100 samples from a large variety of different tumour subtypes, including benign neoplasms, and found that hypermethylation was a general feature of malignancy. More recently, Nelkin et al (1991) found methylation changes to be associated with the accelerated but not the chronic phase of chronic myelogenous leukaemia, suggesting a role for DNA methylation in the later stages of this particular tumour.

We used the ovarian model of tumour development to further examine the association between alterations in DNA methylation

*Received 11 April 1996*
*Revised 16 August 1996*
*Accepted 28 August 1996*

*Correspondence to:* L Dubeau, USC/Norris Comprehensive Cancer Center, 1441 Eastlake Avenue, Los Angeles, CA 90033-0800, USA

*These two authors are listed alphabetically and contributed equally to this work and specific stages of tumorigenesis. Like colorectal tumours, ovarian epithelial tumours are subdivided into benign (cystadenomas) and malignant (carcinomas) categories. In contrast to the benign colorectal tumours that are often regarded as premalignant lesions with a high tendency to progress to carcinomas, the benign ovarian lesions appear more stable and much less likely to undergo such progression. These lesions are therefore presumably more homogeneous as a result of this apparent stability. The attraction of the ovarian model is further enhanced by the fact that these tumours also include a third category, called tumours of low malignant potential (LMP), which are intermediate between cystadenomas and carcinomas and, like the benign tumours, are stable over time.

We examined and compared the levels of DNA methylation in ovarian cystadenomas, LMP tumours and carcinomas in the present manuscript. We reasoned that cystadenomas and LMP tumours could be regarded as early steps of malignant transformation and we therefore sought to determine which step, if any, was associated with DNA methylation changes. We also reasoned that these experiments could provide us with an opportunity to examine whether cystadenomas and LMP tumours are mechanistically similar or not, as the merit of classifying these two tumour subtypes into separate categories is currently the subject of much debate among pathologists (Kurman and Trimble, 1993). The degree of global DNA methylation in ovarian cystadenomas, LMP tumours and carcinomas was compared using high-pressure liquid chromatography (HPLC). We also examined the extent of methylation of a specific gene, called *Myo*D1 in each tumour type. The latter gene was selected for two reasons. First, changes in the patterns of methylation of *Myo*D1 have been reported in various tumour types (Rideout et al, 1994). We speculated that similar changes may be present in ovarian tumours. Second, in contrast to global levels of DNA methylation, which are decreased in most neoplastic tumours (Gama-Sosa et al, 1983), *Myo*D1 methylation is increased in some malignant tumours (Rideout et al, 1994). We were interested to know if changes leading to such hypermethylation of specific genes occurred at the same stages of ovarian tumorigenesis as those leading to global hypomethylation. As we realized that differences in DNA methylation patterns distinguished carcinomas and LMP tumours from cystadenomas, we also sought to verify that ovarian cystadenomas are authentic neoplasms and not hyperplastic conditions in order to better understand the significance of DNA methylation differences between cystadenomas and other subtypes of ovarian epithelial tumours.

### MATERIALS AND METHODS

### Source and processing of tissue specimens

Tumour specimens were obtained fresh from the operating rooms of either the USC/Norris Comprehensive Cancer Center or the Women's Hospital of the Los Angeles County Medical Center and frozen immediately. All tissues were obtained in compliance with the rules of the Institutional Tissue Committee at the University of Southern California and after approval was obtained from that committee. Diagnostic verification of each case was done by one of us (LD), who is a surgical pathologist familiar with ovarian tumour histopathology. Histological sections of all frozen carcinoma and LMP tumour samples were examined before DNA extraction in order to confirm the presence of tumour tissue and to rule out the presence of unacceptable amounts of non-neoplastic stroma. For cystadenomas, the inner linings of the neoplastic cysts were scraped with scalpel blades in order to separate the neoplastic cells from underlying cyst walls. Analysis of cells recovered in such scrapings by flow cytometry revealed that over 99% stained positively with anti-keratin monoclonal antibodies and were therefore of epithelial origin (not shown). DNA was extracted from each tissue sample as described previously (Zheng et al, 1995).

### High-pressure liquid-chromatography

DNA samples were digested by nuclease P1 (Boehringer Mannheim, Indianapolis, IN, USA) and alkaline phosphatase (Promega, Madison, WI, USA) for 4 h at 37°C (Gehrke et al, 1984). Samples were centrifuged, and 25- to 200-µl aliquots of the supernatants were injected directly into a HPLC apparatus (purchased from Waters, Milford, MA, USA), equipped with a 440 Absorbance Detector and a 10 cm Brownlee RP-18 column guarded by a Brownlee Aquaphore ODS precolumn. A solution containing 50 mM sodium dihydrogen phosphate, pH 4.0 and 2.5% methanol was used as mobile phase. The flow rate was set at 1 ml $min^{-1}$, and the absorbance was monitored at 254 nm. Retention times were 6 min for deoxycytosine, 7.4 min for 5-methyldeoxycytosine, 9 min for deoxythymidine, 10.5 min for deoxyguanosine, and 29 min for deoxyadenosine under those conditions. The purity of each peak was verified by variations of mobile phase pH and methanol concentrations. Standard deoxynucleosides were obtained from Sigma (St Louis, MO, USA).

### Southern blotting analysis

An aliquot of 10 µg of each DNA sample was digested with either the methylation-sensitive *Hpa*II or the methylation-insensitive *Msp*I restriction endonuclease following manufacturer's (Boehringer-Mannheim) recommendations. The digested DNA samples were electrophoresed on 1% agarose and transferred to Zetabind nylon membranes (BioRad, Richmond, CA, USA) as described previously (Ehlen and Dubeau, 1990). The membranes were hybridized to a radiolabelled 800-base pair cDNA probe for the *Myo*D1 locus obtained from Professor HH Arnold (Braun et al, 1994) of the University of Braunschweig (Germany), and autoradiographed. Conditions for hybridization and probe labelling were described previously (Ehlen and Dubeau, 1990).

### Analysis of X-chromosome Inactivation

An aliquot of 1 µl of DNA isolated from either the patient's blood or from the epithelial cells lining ovarian cysts was incubated overnight in the presence or absence of 5 units of *Hpa*II restriction endonuclease in a total volume of 10 µl. Polymerase chain reaction (PCR) reagents were then added to each tube, bringing the final volume to 100 µl. Sense and antisense primers were 5′-TGCGCGAAGTGATCCAGAAC-3′ and 5′-CTTGGGGAGAACCATCCTCA-3′ respectively. These primers flank a trinucleotide repeat polymorphism in exon 1 of the androgen receptor gene (Sleddens et al, 1992) as well as two *Hpa*II restriction endonuclease sites located within 100 bases of this polymorphism (Allen et al, 1992). Conditions for the PCR reactions were as follows: 94°C for 30 s, 55°C for 30 s and 72°C for 30 s. PCR was continued for 25 cycles. An aliquot of 1 µl from each reaction was then transferred to a new PCR mix and reamplified with nested primers in a 25-µl reaction volume containing 1 µCi of 2–10 Ci $mmol^{-1}$

© Cancer Research Campaign 1997

**Table 1** 5-Methylcytosine content in normal and neoplastic ovaries

| Tissue source | 5-Methylcytosine (mol %)[a] | n[b] |
|---|---|---|
| Cystadenomas | 0.97 ± 0.08 | 7 |
| LMP tumours | 0.80 ± 0.07 | 8 |
| Carcinomas | 0.73 ± 0.08 | 10 |
| | two-sided $P = 0.0001$[c] | |

[a]Mean ± standard deviation. [b]Number of specimens analysed. [c]The one-way variance method was used to compare levels of 5-methylcytosine between the three groups of ovarian tumours. Subsequent pairwise comparisons showed statistically significant differences between cystadenomas *vs* LMP tumours and cystadenomas *vs* carcinomas. No differences were seen between LMP tumours *vs* carcinomas.

[α-$^{32}$P]dCTP (ICN Radiochemicals, Irvine, CA, USA). Primers for this reamplification reaction were: 5′-GAAGATTCAGC-CAAGCTCAA-3′ and 5′-TGAAGGTTGCTGTTCCTCAT-3′. The radiolabelled products were electrophoresed on 6% polyacrylamide gels under denaturing conditions and autoradiographed.

### Statistical analysis

The analysis of variance method was used to compare 5-methylcytosine levels in cystadenomas, LMP tumours and carcinomas (Dixon and Massey, 1969). Fisher's exact test (Dixon and Massey, 1969) was used to compare the proportions of tumours with methylation changes in the cystadenoma group *vs* the group of LMP tumours and carcinomas. All *P*-values quoted are two-sided.

### RESULTS

### Global levels of methylation in ovarian cystadenomas, LMP tumours and carcinomas

We measured the global levels of methylation in DNA samples obtained from seven ovarian cystadenomas, eight LMP tumours and ten carcinomas in order to determine if these different tumour subtypes, which can be regarded as representing different degrees of neoplastic transformation, could be distinguished on the basis of such measurements. DNA isolated from the various ovarian tumours was digested to single nucleosides and analysed by HPLC as explained in Materials and methods. This approach gives an accurate determination of the percentage of methylated cytosine residues, as incompletely digested products or impurities are readily identified as extra peaks in the chromatogram. Mean global levels of DNA methylation showed significant differences ($P = 0.0001$) among the three ovarian tumour subtypes (Table 1). Subsequent pairwise comparisons revealed significant differences in mean levels of 5-methylcytosine between cystadenomas and LMP tumours and between cystadenomas and carcinomas. Mean levels in LMP tumours and carcinomas were 79% and 75% respectively of the mean in cystadenomas. There was no difference in mean levels of 5-methylcytosine content between LMP tumours and carcinomas (Table 1).

The above seven cystadenomas included two tumours with serous differentiation and five mucinous tumours. Five of the eight LMP tumours were serous whereas the remaining three were mucinous. Eight of the ten carcinomas were serous whereas two were endometrioid. Accurate comparisons between these different histological subtypes within each category is not possible because

**Figure 1** Methylation of the *MyoD*1 locus in ovarian cystadenomas and LMP tumours. DNA obtained from either cystadenomas or LMP tumours was digested with *Msp*I (M) or *Hpa*II (H) restriction endonucleases and analysed by Southern blotting using a radiolabelled probe for the *MyoD*1 locus. The figure shows autoradiographs of representative Southern blots. The numbers on the left of each autoradiograph indicate fragment lengths (base pairs)

© Cancer Research Campaign 1997

**Table 2** Methylation of *Hpa*II restriction endonuclease sites in normal and neoplastic ovarian tissues

| Tissue source | Cases with no methylation | Cases with methylation | Proportion of cases with methylation |
|---|---|---|---|
| Normal ovaries | 6, 14, 17, 18, 19 | None | 0/5 |
| Cystadenomas | 3, 4, 5, 6, 11, 12, 15, 37, 47, 50 | None | 0/10[a] |
| LMP tumors | 1, 6, 7, 10, 11 | 3, 5, 31, 39, 40 | 5/10[a] |
| Carcinomas | 31, 35, 40, 67, 69 | 41, 43, 47, 61, 64 | 5/10 |

[a]The proportion of cystadenomas with methylation was significantly different from that of LMP tumors (two-sided $P = 0.03$).
Tumour histological subtypes: serous cystadenomas (nos 5, 6, 11, 37), mucinous cystadenomas (nos 3, 4, 12, 15, 50), simple cystadenoma (no. 47), serous LMP tumours (nos 1, 3, 7, 10, 39, 40), mucinous LMP tumours (nos 5, 6, 11, 31), serous carcinomas (nos 31, 35, 41, 43, 47, 61, 67, 69), endometrioid carcinomas (40, 64).

**Figure 2** Clonal origin of ovarian cystadenomas. DNA obtained from either blood (B) or tumour (T) samples of a patient with a large serous ovarian cystadenoma was either left undigested or digested with the *Hpa*II restriction endonuclease. Each sample was then amplified enzymatically using primers flanking a trinucleotide repeat polymorphism in the first exon of the androgen receptor gene as well as two *Hpa*II sites located within 100 base pairs of this polymorphism. The radiolabelled PCR products were electrophoresed on 6% agarose under denaturing conditions and autoradiographed of the small number of tumours examined. However, there were no apparent associations between DNA methylation levels and either serous, mucinous, or endometrioid differentiation.

We also measured 5-methylcytosine levels in four samples of normal ovarian tissues. Such levels were significantly higher than in cystadenomas (1.51 mol%, standard deviation 0.19%). The significance of these differences between normal ovarian tissues and cystadenomas, however, is unclear (see Discussion).

### Methylation of the *Myo*D1 locus in ovarian cystadenomas, LMP tumours and carcinomas

The above results suggest that global changes in DNA methylation in ovarian epithelial tumours are most extensive in LMP tumours and carcinomas. We therefore assessed levels of methylation of the *Myo*D1 locus in such tumours in order to determine whether methylation changes also correlate with the degree of neoplastic transformation at the level of individual genes. Interestingly, studies of *Myo*D1 methylation in other tumour models suggested that there is increased methylation at this locus in malignant tumours (Rideout et al, 1994), reflecting the fact that both, local hypo- and hypermethylation changes occur during tumorigenesis. We therefore sought to determine if changes affecting the *Myo*D1 locus occur at the same stage of tumorigenesis as those responsible for generalized genomic hypomethylation. We took advantage of the fact that two potential methylation sites in the *Myo*D1 locus are within recognition sequences for the methylation-sensitive *Hpa*II restriction endonuclease. Methylation of these two sites has previously been associated with malignant transformation (Rideout et al, 1994). DNA from the various tumour samples was digested either with this enzyme or with the *Msp*I endonuclease. The latter enzyme recognizes the same sequence as *Hpa*II but is methylation insensitive. The digested samples were electrophoresed on 1% agarose and analysed by Southern blotting using a cDNA probe for *Myo*D1.

The results for selected cystadenomas and LMP tumours are shown in Figure 1. DNA digested with *Msp*I produced a single fragment of 603 base pairs in each case. The same fragment was obtained after digestion of cystadenoma DNA with *Hpa*II. Thus, the *Hpa*II sites at the *Myo*D1 locus were totally unmethylated in these tumours because the presence of methylated cytosines would have prevented cleavage by this enzyme and resulted in fragments of larger sizes. Such larger fragments were found in three of the five LMP tumours illustrated in the figure.

The above approach was used to examine the methylation status of *Hpa*II sites in the *Myo*D1 locus in five normal ovaries, ten cystadenomas, ten LMP tumours and ten carcinomas. The results are shown in Table 2. None of the five normal ovarian tissue samples showed methylation of these sites. Likewise, DNA methylation was not detected in any of the ten cystadenomas examined. In contrast, five of the ten LMP tumours and five of the ten carcinomas showed methylation of either one or both *Hpa*II sites (Table 2). We compared the proportion of cystadenomas showing methylation changes with that of LMP tumours and found the difference to be statistically significant ($P = 0.03$).

### Ovarian cystadenomas are authentic neoplasms

We examined eight ovarian cystadenomas ranging in size from 3 to 12 cm and determined whether these tumours were of monoclonal origin or not. Our intention was to better understand the significance of the distribution of alterations in DNA methylation in ovarian tumours, as it was unclear to us if ovarian cystadenomas represented neoplastic or hyperplastic lesions (see Discussion). Monoclonality is a feature of neoplasia whereas polyclonality is usually indicative of non-neoplastic disorders such as hyperplasia.

© Cancer Research Campaign 1997

We therefore used an approach first developed by Vogelstein et al (1985) to examine the monoclonal origin of the above eight cystadenomas. This approach is based on the fact that all tumour cells within a given tumour mass show inactivation of the same allele of the X chromosome if the tumour originated from a single cell (monoclonal). In contrast, polyclonal cell populations show random inactivation of one or the other X chromosome allele. DNA from each of the above tumour samples as well as from each patient's blood was either undigested or digested with the *Hpa*II restriction enzyme. This enzyme is methylation sensitive and therefore does not cleave DNA at methylated sites. The digested fragments were amplified enzymatically using primers flanking a trinucleotide repeat polymorphism in the androgen receptor gene on chromosome Xq (Sleddens et al, 1992). These primers also flanked two sites for the *Hpa*II restriction endonuclease that are adjacent to the trinucleotide repeat. At least one of these sites is unmethylated if it is located on an active allele of the X chromosome whereas both sites are invariably methylated if they are located on an inactive allele (Allen et al, 1992). Thus, only sequences from inactive X chromosome alleles are amplifiable using the above set of primers because sequences from active alleles, which are sensitive to *Hpa*II digestion, are cleaved during the prior digestion with this enzyme.

The results of a representative experiment are shown in Figure 2. Undigested DNA from either blood or tumour samples showed fragments of two different sizes after enzymatic amplification with the above primers (Figure 2). These two fragments corresponded to the two alleles of the androgen receptor gene in this patient. Both alleles were also present in digested DNA from the patient's blood. This indicates that an equal proportion of each allele was resistant to *Hpa*II digestion in this sample, confirming a polyclonal origin. However, only one of the two alleles could be amplified in digested tumour DNA, indicating that X inactivation affected this allele exclusively in all tumour cells from this patient. Similar results were obtained with all remaining seven cases examined (not shown). We conclude that ovarian cystadenomas are monoclonal and are therefore true neoplasms.

### DISCUSSION

The time at which alterations in DNA methylation take place during tumour development is still not clear. Although such changes are clearly associated with the malignant phenotype in a large number of different tumour types (Gama-Sosa et al, 1983), they are also found in some benign neoplasms such as colorectal polyps (Goelz et al, 1985; Feinberg et al, 1988). Ovarian epithelial tumours are a good model to address this question because there are two well-defined subtypes of these tumours that do not fully express the malignant phenotype (Cheng et al, 1996) and can therefore be regarded as different early stages of malignant transformation. The results of our experiments clearly show that DNA methylation changes are present in both, ovarian LMP tumours and carcinomas, lending further support to the idea advanced by Goelz et al (1985) that such changes are early events in malignant transformation. However, our results also show that global alterations in genomic methylation are less extensive (and perhaps absent) in cystadenomas, which are authentic (although benign) neoplasms based on our demonstration of their monoclonal origin. Changes in methylation of the *Myo*D1 locus, which were similar in LMP tumours and carcinomas, were likewise not detected in cystadenomas. We conclude that alterations in DNA methylation are early, but not initial events in ovarian tumorigenesis because they occur in LMP tumours, the phenotypic manifestations of which are intermediate between fully malignant and clearly benign ovarian tumours.

The above results also emphasize the merit of subdividing the non-invasive and non-metastatic subtypes of ovarian tumours into cystadenomas and LMP tumours. This issue is the subject of much debate among pathologists. Indeed, it has been argued that LMP tumours (at least those showing serous differentiation) typically behave as benign lesions clinically and should not therefore be regarded as a separate disease entity (Kurman and Trimble, 1993). However, our results suggest that such tumours (three of the five LMP tumours with methylation changes at the *Myo*D1 locus were of serous differentiation) may develop through different genetic mechanisms from cystadenomas. This conclusion is also supported by recent data from our laboratory (Cheng et al, 1996). The available evidence therefore supports the notion that LMP tumours are mechanistically more complex than cystadenomas and strongly argues in favour of maintaining the distinction between these two variants of ovarian epithelial tumours, in spite of the fact that they often show similar clinical behaviour.

We also observed substantial differences between levels of methylation in cystadenomas and those present in normal ovarian tissues. It is possible that these differences reflect methylation changes associated with the development of ovarian cystadenomas. However, normal ovaries are made up of a variety of different cell types including stromal cells, germ cells, follicular cells, etc. These different cell types probably show significant differences in their levels and patterns of DNA methylation because of their widely different functions and spectra of gene expression. Ovarian surface epithelial cells, which are the alleged origin of ovarian epithelial tumours, account for less than 1% of the cell types present in normal ovarian tissue samples. Thus, we favour the explanation that the observed differences between the methylation levels of normal ovaries compared with ovarian cystadenomas are due primarily to differences in the various cell types present in those tissue samples.

A widely accepted theory stipulates that ovarian cystadenomas arise from invaginations of the ovarian surface, resulting in entrapment of the surface epithelial cells which, as they continue to proliferate, form intra-ovarian cysts. If true, the initial steps in cystadenoma development are not neoplastic and the above mechanism could be compatible with a hyperplastic phenomenon. Indeed, small (<1 cm) ovarian cysts are regarded as non-neoplastic by convention. We considered the possibility that larger cysts were also non-neoplastic because the differences in the methylation levels of cystadenomas compared to LMP tumours and carcinomas suggest fundamentally different underlying mechanisms for these tumour subtypes. We therefore determined whether cystadenomas were monoclonal or polyclonal because most neoplasms are thought to have a monoclonal origin whereas hyperplasias are thought to be polyclonal. However, our finding that ovarian cystadenomas are monoclonal provides strong support for their alleged neoplastic nature and does not support a hyperplastic origin. Thus, the clear differences in the DNA methylation levels of LMP tumours or carcinomas when compared with cystadenomas suggest that, although methylation changes are a general feature of malignant neoplasms, such changes are not essential for neoplastic transformation per se, at least in ovarian tumours.

It is not known if the above alterations in DNA methylation are causes or consequences of ovarian carcinoma and LMP tumour development. However, the fact that the expression of genes

*© Cancer Research Campaign 1997*

involved in growth control and tumorigenesis was reported to be altered as a result of DNA methylation changes in tumours of different histological types suggests that such alterations may indeed play an active and important role in the establishment of the malignant phenotype. Possibilities include the silencing of tumour-suppressor genes or the increased expression of proto-oncogenes (Balmain, 1995). Identification of specific genetic targets for methylation changes in ovarian epithelial tumours may not only lead to a better understanding of the molecular mechanisms and determinants of their development, but may also facilitate the use and monitoring of methylation-targeting drugs in the treatment of ovarian cancer patients.

### ABBREVIATIONS

LMP, low malignant potential; HPLC, high-pressure liquid chromatography.

### ACKNOWLEDGEMENTS

This work was supported by grants R01 CA51167, R01 CA60743 and R35 CA53890 from the National Cancer Institute and by grant CN75327 from the American Cancer Society. Christoph Schmutte is a post doctoral fellow in the laboratory of Dr Peter A Jones at USC and was supported by grant R35 CA49758 from the National Cancer Institute. We thank Kazuko Arakawa for assistance in data analysis.

### REFERENCES

Allen RC, Zoghbi HY, Moseley AB, Rosenblatt HM and Belmont JW (1992) Methylation of *Hpa*II and *Hha*I sites near the polymorphic CAG repeat in the human androgen-receptor gene correlates with X chromosome inactivation. *Am J Hum Genet* **51**: 1229–1239

Antequera F, Boyes J and Bird A (1990) High levels of *de novo* methylation and altered chromatin structure at CpG islands in cell lines. *Cell* **62**: 503–514

Balmain A (1995) Exploring the bowels of DNA methylation. *Curr Biol* **5**: 1013–1016

Bhave MR, Wilson MJ and Poirier LA (1988) c-H-ras and c-K-ras gene hypomethylation in the livers and hepatomas of rats fed methyl-deficient, amino acid-defined diets. *Carcinogenesis* **9**: 343–348

Bird AP (1986) CpG-rich islands and the function of DNA methylation. *Nature* **321**: 209–213

Bird AP (1992) The essentials of DNA methylation. *Cell* **70**: 5–8

Braun T, Bober E, Rudnicki MA, Jaenisch R and Arnold HH (1994) MyoD expression marks the onset of skeletal myogenesis in myf-5 mutant mice. *Development* **120**: 3083–3092

Cheng PC, Gosewehr J, Kim TM, Velicescu M, Wan M, Zheng J, Felix JC, Cofer KF, Luo P, Biela B, Godorov G and Dubeau L (1996) Potential role of the inactivated X chromosome in ovarian epithelial tumor development. *J Natl Cancer Inst* **88**: 510–518

Dixon WJ and Massey FJ (1969) *Introduction to Statistical Analysis*, 3rd ed. McGraw-Hill Book Company: New York

Ehlen T and Dubeau L (1990) Loss of heterozygosity on chromosomal segments 3p, 6q and 11p in human ovarian carcinomas. *Oncogene* **5**: 219–223

Ehrlich M and Wang RY (1981) 5-Methylcytosine in eukaryotic DNA. *Science* **212**: 1350–1357

Fearon ER and Vogelstein B (1990) A genetic model for colorectal tumorigenesis. *Cell* **61**: 759–767

Feinberg AP and Vogelstein B (1983) Hypomethylation distinguishes genes of some human cancers from their normal counterparts. *Nature* **301**: 89–92

Feinberg AP, Gehrke CW, Kuo KC and Ehrlich M (1988) Reduced genomic 5-methylcytosine content in human colonic neoplasia. *Cancer Res* **48**: 1159–1161

Felgner J, Kreipe H, Heidorn K, Jaquet K, Zschunke F, Radzun HJ and Parwaresch MR (1991) Increased methylation of the c-fms proto-oncogene in acute myelomonocytic leukemias. *Pathobiology* **59**: 293–298

Gama-Sosa MA, Slagel VA, Trewyn RW, Oxenhandler R, Kuo KC, Gehrke CW and Ehrlich M (1983) The 5-methylcytosine content of DNA from human tumors. *Nucleic Acids Res* **11**: 6883–6894

Gehrke CW, Mccune RA, Gama-Sosa M, Ehrlich M and Kuo KC (1984) Quantitative reversed-phase high-performance liquid chromatography of major and modified nucleosides in DNA. *J Chromatography* **301**: 199–219

Goelz SE, Vogelstein B, Hamilton SR and Feinberg AP (1985) Hypomethylation of DNA from benign and malignant human colon neoplasms. *Science* **228**: 187–190

Gonzalez-Zulueta M, Bender CM, Yang AS, Nguyen TT, Beart RW, van Tornout JM and Jones PA (1995) Methylation of the 5′ CpG island of the p16/CDKN2 tumor suppressor gene in normal and transformed human tissues correlates with gene silencing. *Cancer Res* **55**: 4531–4535

Greger V, Passarge E, Hopping W, Messmer E and Horsthemke B (1989) Epigenetic changes may contribute to the formation and spontaneous regression of retinoblastoma. *Hum Genet* **83**: 155–158

Hanada M, Delia D, Aello A, Stadtmauer E and Reed JC (1993) Bcl-2 gene hypomethylation and high-level expression in B-cell chronic lymphocytic leukemia. *Blood* **82**: 1820–1828

Herman JG, Latif F, Weng Y, Lerman MI, Zbar B, Liu S, Samid D, Duan DS, Gnarra JR and Linehan WM and Baylin SB (1994) Silencing of the VHL tumor-suppressor gene by DNA methylation in renal carcinoma. *Proc Natl Acad Sci USA* **91**: 9700–9704

Jones PA, Wolkowicz MJ, Rideout WM, Gonzalez FA, Marziasz CM, Coetzee GA and Tapscott SJ (1990) *De novo* methylation of the MyoD CpG island during the establishment of immortal cell lines. *Proc Natl Acad Sci USA* **87**: 6117–6121

Keshet I, Yisraeli J and Cedar H (1985) Effect of regional DNA methylation on gene expression. *Proc Natl Acad Sci USA* **82**: 2560–2564

Kurman RJ and Trimble CL (1993) The behavior of serous tumors of low malignant potential: are they ever malignant? *Int J Gynecol Pathol* **12**: 120–127

Laird PW and Jaenisch R (1994) DNA methylation and cancer. *Hum Mol Genet* **3**: 1487–1495

Li E, Bestor T-H and Jaenisch R (1992) Targeted mutation of the DNA methyltransferase gene results in embryonic lethality. *Cell* **69**: 915–926

Li E, Beard C and Jaenisch R (1993) Role for DNA methylation in genomic imprinting. *Nature* **366**: 362–365

Lipsanen V, Leinonen P, Alhonen L and Janne J (1988) Hypomethylation of ornithine decarboxylase gene and erb-A1 oncogene in human chronic lymphatic leukemia. *Blood* **72**: 2042–2044

Makos M, Nelkin BD, Reiter RE, Gnarra JR, Brooks J, Isaacs W, Linehan M and Baylin SB (1993) Regional DNA hypermethylation at D17S5 precedes 17p structural changes in the progression of renal tumors. *Cancer Res* **53**: 2719–2722

Merlo A, Herman JG, Mao L, Lee DJ, Gabrielson E, Burger P, Baylin SB and Sidransky D (1995) CpG island methylation is associated with transcriptional silencing of the tumor suppressor p16/CDKN2/MTS1 in human cancers. *Nature Med* **1**: 686–692

Mohandas T, Sparkes RS and Shapiro LJ (1981) Reactivation of an inactive human X chromosome: evidence for X inactivation by DNA methylation. *Science* **211**: 393–396

Munzel PA, Pfohl LA, Rohrdanz E, Keith G, Dirheimer G and Bock KW (1991) Site-specific hypomethylation of c-*myc* proto-oncogene in liver nodules and inhibition of DNA methylation by *N*-nitrosomorpholine. *Biochem Pharmacol* **42**: 365–371

Nelkin BD, Przepiorka D, Burke PJ, Thomas ED and Baylin SB (1991) Abnormal methylation of the calcitonin gene marks progression of chronic myelogenous leukemia. *Blood* **77**: 2431–2434

Ray JS, Harbison ML, McClain RM and Goodman JI (1994) Alterations in the methylation status and expression of the raf oncogene in phenobarbital-induced and spontaneous B6C3F1 mouse live tumors. *Mol Carcinogen* **9**: 155–166

Rideout WM, Eversole-Cire P, Spruck CH, Hustad CM, Coetzee GA, Gonzales F and Jones PA (1994) Progressive increases in the methylation status and heterochromatinization of the myoD CpG island during oncogenic transformation. *Mol Cell Biol* **14**: 6143–6152

Sakai T, Toguchida J, Ohtani N, Yandell DW, Rapaport JM and Dryja TP (1991) Allele-specific hypermethylation of the retinoblastoma tumor-suppressor gene. *Am J Hum Genet* **48**: 880–888

Selig S, Ariel M, Goitein R, Marcus M and Cedar H (1988) Regulation of mouse satellite DNA replication time. *EMBO J* **7**: 419–426

Sharrard RM, Royds JA, Rogers S and Shorthouse AJ (1992) Patterns of methylation of the c-*myc* gene in human colorectal cancer progression. *Br J Cancer* **65**: 667–672

© Cancer Research Campaign 1997

Sleddens HF, Oostra BA, Brinkmann AO and Trapman J (1992) Trinucleotide repeat polymorphism in the androgen receptor gene (AR). *Nucleic Acids Res* **20**: 1427

Spruck CH, Rideout WM and Jones PA (1993) DNA methylation and cancer. In *DNA Methylation: Molecular Biology and Biological Significance*. Jost JP and Saluz HP, (eds) pp. 487–509 Birkhauser: Basle

Vertino PM, Spillare EA, Harris CC and Baylin SB (1993) Altered chromosomal methylation patterns accompany oncogene-induced transformation of human bronchial epithelial cells. *Cancer Res* **53**: 1684–1689

Vogelstein B, Fearon ER, Hamilton SR and Feinberg AP (1985) Use of restriction fragment length polymorphisms to determine the clonal origin of human tumors. *Science* **227**: 642–645

Zheng J, Wan M, Zweizig S, Velicescu M, Yu MC and Dubeau L (1993) Histologically benign and low grade malignant tumors adjacent to high grade ovarian carcinomas are not pre-existing precursor lesions. *Cancer Res* **53**: 4138–4142

Zheng JP, Benedict WF, Xu H-J, Hu S-X, Kim TM, Velicescu M, Wan MH, Cofer KF and Dubeau L (1995) Genetic disparity between morphologically benign cysts contiguous to ovarian carcinomas acid solitary cystadenomas. *J Natl Cancer Inst* **87**: 1146–1153

© Cancer Research Campaign 1997